# MF-Storm: a maximum flow-based job scheduler for stream processing engines on computational clusters to increase throughput

**DOI:** 10.7717/peerj-cs.1077

**Published:** 2022-09-26

**Authors:** Asif Muhammad, Muhammad Abdul Qadir

**Affiliations:** Department of Computer Science, Capital University of Science & Technology, Islamabad, Punjab, Pakistan

**Keywords:** Stream processing engines, Resource-aware, Heterogeneous cluster, Job scheduler, APACHE storm

## Abstract

**Background:**

A scheduling algorithm tries to schedule multiple computational tasks on a cluster of multiple computing nodes to maximize throughput with optimal utilization of computational and communicational resources. A Stream Processing Engine (SPE) is deployed to run streaming applications (computational tasks) on a computational cluster which helps execution and coordination of these applications. It is observed that there is a gap in the optimal mapping of a computational and communicational load of a streaming application on the underlying computational and communication power of the resources (cluster). Frequently communicated tasks are scheduled at different processing nodes with relatively slow communicating links. This increases network latency with a decrease in resource utilization. Hence, reduces the achieved throughput of the cluster significantly.

**Methods:**

MF-Storm, a max-flow min-cut based job scheduler is presented to achieve a near-optimum schedule to maximize throughput. It schedules a streaming application by considering the processing, communication demands, available computational and communicational resources in a heterogeneous cluster, dynamically with minimized scheduling cost. To keep the scheduling cost minimum, the scheduler is built in a pipeline with two major stages: in the first stage, the application’s tasks graph is partitioned using the max-flow min-cut algorithm to minimize inter-partition traffic, and in the second stage, these partitions are assigned to computing nodes according to the computational power of the cluster’s nodes.

**Results:**

Extensive experiments were done to evaluate the performance of MF-Storm using different topologies with multiple scenarios on a physical cluster implementation. Results showed on average 148% improvement in throughput with 30% less computational resources as compared to different state-of-the-art schedulers.

## Introduction

A data stream is a continuous flow of data that needs to be processed by computing applications. This data might be sensor data, network data, stock prices, postings on social networks, *etc.* Processing these data streams in real-time demands ever-increasing processing power. Multiple machines combined in a distributed fashion to process the data is an economical way to solve the problem. In a cluster of distributed computing elements, Stream Processing Engines (SPEs) (Liu & Buyya, 2020) have been formulated to process such data streams in real-time. To address the ever-increasing needs of the industry, SPEs have rapidly evolved in the last two decades with several SPE prototypes being adopted by the industry, for example, Apache Storm (Storm A, 2014), Apache Spark Streaming (Foundation AS, 2018), Apache Flink (Foundation AS, Apache Software Foundation, 2015), and Apache Heron (Twitter, 2014), *etc.* One of the important research issues for SPE is how to schedule the processing tasks on processing nodes to process the streams with near-optimum use of the available resources with maximized throughput. The scheduling policy of SPEs on clusters of multiple machines decides how tasks are distributed in the stream processing system. Typically, SPEs are assessed based on communication latency or system throughput (Falt & Yaghob, 2011). In the context of SPEs, throughput refers to the average number of jobs per time unit (Tantalaki, Souravlas & Roumeliotis, 2020). In contrast, latency refers to the elapsed time from job submission to receiving the first response after processing the job (Tantalaki, Souravlas & Roumeliotis, 2020). Stream processing consumes multiple resources *e.g*., CPU cycles, memory, network bandwidth, disk I/O, *etc.* An efficient scheduler assigns the tasks in a manner that minimizes task completion time and increases the utilization of resources (Tantalaki, Souravlas & Roumeliotis, 2020).

A data stream application can be modeled as *Directed Acyclic Graph* (DAG) which represents the processing tasks and communication between these tasks required to complete the DSP application (Kamburugamuve & Fox, 2016). This abstraction layer helps a scheduler to understand how to place these tasks on multiple processing elements to achieve the objectives (Cardellini et al., 2018). To schedule these tasks optimally, graph partitioning algorithms are being used in research (Fischer & Bernstein, 2015; Sun et al., 2015; Eskandari, Huang & Eyers, 2016; Ghaderi, Shakkottai & Srikant, 2016; Li & Zhang, 2017; Eskandari et al., 2018a, 2018b; Ullah et al., 2019; Gulzar Ahmad et al., 2020) to place frequently communicating components of the DAG closer to each other to reduce latency which may improve overall throughput, too. But most of them (Fischer & Bernstein, 2015; Liu, 2017; Liu et al., 2017; Qian et al., 2017; Tahir et al., 2019; Gulzar Ahmad et al., 2020) are either resource-unaware or non-adaptive. As a result, it is difficult to decide on the power of cluster resources. Similarly, some techniques (Fischer & Bernstein, 2015; Smirnov, Melnik & Nasonov, 2017; Weng et al., 2017; Gulzar Ahmad et al., 2020; Elahi et al., 2022) are proposed for homogenous clusters only and are unable to assign workload according to the node's computing power (heterogeneity). In recent years, resource-aware schedulers (Peng et al., 2015; Xue et al., 2015; Weng et al., 2017; Liu & Buyya, 2018; Eskandari et al., 2018b; Al-Sinayyid & Zhu, 2020; Muhammad & Aleem, 2021; Muhammad, Aleem & Islam, 2021; Farooq et al., 2022) are also proposed which are either non-adaptive or static in nature and cannot accommodate real-time (dynamic) changes. Moreover, CPU, Memory, and bandwidth are considered a resource by existing resource-aware schedulers, however, they do not truly represent the computation power of a node (“FLOPS (Floating Point Operations Per Second) Definition”, https://techterms.com/definition/flops).

To address these questions, we present MF-Storm—A maximum flow-based job scheduler for stream processing engine (Apache Storm) which assigns frequently communicating DAG components closer to each other such that minimum nodes are employed for job execution.

The main contribution of this article is the following:

- MF-Storm–An adaptive scheduler that employs the topology mapping using max-flow min-cut algorithm (“Max-flow min-cut theorem—Wikipedia”, https://en.wikipedia.org/wiki/Max-flow_min-cut_theorem) such that frequently communicating executors are placed closely to maximize throughput;
- MF-Storm is also a resource-aware scheduler that assigns components to machines based on their computational power;
- Performance assessment with state-of-the-art schedulers showed that MF-Storm increases average throughput up to 148% while using 30% less computational resources.

The rest of the article is organized as follows: “Literature review” consists of the research work done in this domain with their limitations. “MF-Storm scheduler” explains MF-Storm with the methodology adopted to do experimentation. “Experimental evaluation” defines the experimental setup used followed by performance evaluation. Results are compared with the latest schedulers and are also debated in this section. “Conclusions and future work” concludes the article with future directions.

## Literature review

To improve the throughput and resource utilization of SPEs, different researchers have presented their work in this domain. In 2014, Xu et al. (2014) proposed a traffic-aware scheduler (called T-Storm) which uses runtime states to accelerate data processing for dynamic task allocation. This minimizes inter-process traffic in a load-balancing fashion. T-Storm was implemented using Storm 0.8.2. Experiments show up to 84% improvement in average throughput with 30% less computing nodes as compared to the default scheduler. However, the proposed work faces the cold start problem which means that initially some data is needed for this type of scheduler to run.

van der Veen et al. (2015) have also proposed a scheduler for Apache Storm with a different methodology. First, resource over-provisioning or under-provisioning is detected. The automatic addition or removal of nodes is performed for under-provisioned or over-provisioning of the resources. The major drawback of this work is that it ignores network structure while assigning tasks to a cluster. Due to this, equal tasks assignment is performed despite their physical distance which may introduce latency. The proposed methodology is compared with Storm version 0.9.2 and experiments are carried out on a cluster of one master node, one zookeeper node, and several processing nodes.

Due to heterogeneous clusters, increasing the number of nodes for a job execution does not guarantee the overall performance improvement. This problem is addressed by Xue et al. (2015) in two different scenarios. For systems using a hash-based partition method, a greedy approach is used to select suitable workers for job execution. For systems that allow arbitrary graph partition, a heterogeneity-aware graph partitioning model is presented that assigns workload at a fine-grained level. The proposed framework reduced the execution time by 55% and 44% for the university’s cluster and Amazon EC2 cluster, respectively. Social network graphs were employed in the experiments and comparison was performed with PageRank, random walk, shortest path, *etc.*

How to distribute workload to available machines strongly affects the overall performance of the stream processing system. To answer this question, Fischer & Bernstein (2015) presented a workload scheduler based on a graph partitioning algorithm. The proposed scheduler collects the communication behaviour of running applications and creates the schedules by partitioning the resulting communication graph using the METIS graph partitioning software. The proposed scheduler showed better performance with decreases of network bandwidth up to 88% and increased throughput values of 56%, respectively. The major limitation of the proposed approach is that it ignores the computational resources while scheduling. Experimental evaluation was performed on a cluster of 80 machines and a comparison was made with default Storm 0.9.0.1 and Aniello, Baldoni & Querzoni (2013) schedulers using OpenGov, Parallel, Payload, and Reference topologies.

The Storm scheduler is a resource-unaware scheduler. In 2015, Peng et al. (2015) proposed a resource-aware scheduler (R-Storm) within the Storm. R-Storm satisfies resource constraints such as CPU, memory, bandwidth, and node physical distance while scheduling. Due to the static scheduling, it is unaware of the runtime workload and remains unchanged during job execution. PageLoad and processing topologies are used for experimentation on a cluster of 13 machines. Results showed a 50% higher throughput and 350% better CPU utilization when compared to the Storm scheduler.

In Apache Storm, the topology configuration cannot be changed during execution. This stagnant configuration is also unaware of data stream properties like transfer rate *etc.* If the data arrival rate surpasses the system processing capacity then this limitation can degrade the throughput of the system. Madsen & Zhou (2015) addressed this issue by over-provisioning of resources. This over-provisioning result in high resource cost which is not used during execution most of the time. Experiments were carried out on Amazon EC2 (25 instances) using Airline On-Time dataset which reduced latency by 20% as compared to the default scheduler.

In general, communication between a topology’s components influences the overall performance of a topology. While scheduling topology, managing inter-node, and inter-slot communication trade-off has become a challenging task. Fan, Chen & Hu (2016) proposed a scheduler based on runtime statistics as well as cluster workload. 67% improvement in throughput is obtained using 3% extra resources with respect to the Storm scheduler (version 0.9.3) using the SOL benchmark. The cluster employed for evaluation consisted of five blade servers.

The default scheduler equally distributes the executors to the computing nodes having two drawbacks. First, it ignores the communication traffic which results in high latency. Secondly, it engages all the nodes, which may increase inter-executor traffic. Eskandari, Huang & Eyers (2016) proposed P-Scheduler, to address these problems. P-Scheduler first calculates the required resources for topology execution to reduce inter-node traffic. Then, the cluster is consolidated after workload estimation. Finally, it enhanced traffic by placing frequently communicating tasks closely. Experiments were performed using 10 computing nodes resulting in 50% less latency to default Storm 0.9.5. In-house, test throughput and top trending topics topologies were employed in these experiments.

In 2017, Zhang et al. (2017) and Li & Zhang (2017) presented a traffic-aware scheduler based on the node’s workload. First, it assigns tasks to slots according to topology structure and inter-component communication. Second, it picks the least loaded node and assigns slots that require maximum resources. The allocation considers memory, and CPU utilization also. This scheduler is compared with default scheduler 0.8.2, R-Storm, and topology-based schedulers using linear, star, and diamond topologies. The evaluation carried out with 8 nodes showed a 91% improvement in throughput and a 50% reduction in latency.

The SPEs work on continuous real-time data. Smirnov, Melnik & Nasonov (2017) proposed a genetic algorithm-based scheduler for real-time SPEs. The problem is to assign tasks to the best-fit node which minimizes traffic and maximizes resource utilization. The proposed methodology is compared with the default and R-Storm schedulers showed a 40% improvement in throughput.

Weng et al. (2017) presented AdaStorm to solve the static topology configuration issue of the default scheduler. With the help of a machine learning algorithm, AdaStorm dynamically adjusts the topology configuration according to the communication traffic. In this way, the static configuration issue has been solved but it does not consider network structure while scheduling, which may affect the overall throughput. AdaStorm was compared with R-Storm using a cluster of 10 nodes. The GeoLife GPS Trajectories dataset was used in these experiments. Results showed that AdaStorm reduces CPU and memory usage by about 15% and 60% respectively.

The Storm scheduler assigns tasks in a round-robin manner. The cluster heterogeneity introduces a performance bottleneck due to an imbalanced task distribution. To address this issue, Liu (2017) proposed a topology-based scheduler (TOSS). TOSS improves performance by reducing the communication overhead. First, TOSS examines topology structure and partition executors to minimize communication. Then, it utilizes the historical workload to estimate the current workload. TOSS was compared with default Storm scheduler version 0.8.2 which resulted in a 24% boost for throughput and a 20% reduction in latency for SOL and Rolling WordCount topology. A small cluster of five machines was used for experiments.

Apache Storm’s state management is achieved by a checkpointing framework, which commits states regularly. This requires a data store for storage and retrieval of state, resulting in performance overhead. A state management system (E-Storm) is proposed (Liu et al., 2017) to address this issue that maintains multiple backups on different nodes. E-Storm recovery operates at the thread level and is integrated into Storm’s execution flow. The replication of the state allows multiple transfers to occur concurrently. E-storm beats the existing checkpointing technique in application performance, obtaining nine times better throughput while reducing the latency down to 9.8%. These results are achieved using 12 nodes cluster at the expense of storage space for multiple backups and processing required to restore the lost state from a checkpoint. A comparison of E-Storm was made with Apache Storm version 1.0.2.

The default scheduler ignores the dependency relationship among workers. As a result, the load-imbalancing problem may occur either the topology run failed or new nodes are added to the cluster. To address this issue, a slot-aware scheduler (named as S-Storm) is proposed (Qian et al., 2017). First, S-Storm evenly allocates slots for multiple topologies in a load-balancing manner. Second, when load-imbalancing happens, S-Storm distributes workers to slots among light-load computing nodes. S-Storm achieved 18.7% speedup on the average processing time and 1.25 times improvement on throughput for Word count and Throughput test topologies as compared to Storm version 0.10.0.

SPEs cannot adapt the scheduling plan according to the resource consumption of the topologies. To deal with this issue, Liu & Buyya (2018) presented D-Storm which observes executing applications to obtain resource utilization and communication history. To reduce inter-node traffic, run-time decisions are made to schedule tasks closer to each other. In case of resource contention, a new scheduling plan is generated automatically. Despite these features, D-Storm is suffered from a cold start problem. Similarly, D-Storm is 20 times slower in generating an execution plan as compared to the default scheduler 1.0.2. Performance evaluation was performed on Nectar Cloud (using 19 nodes) with the default scheduler and R-Storm. D-Storm achieved a 16.25% improvement on throughput for Tweet sentiment topology.

Eskandari et al. (2018b) proposed T3-Scheduler based on Apache Storm 1.1.1, which finds frequently communicating tasks and allocates them closer while making sure that each node is fully utilized. First, it divides topology based on communication patterns into multiple parts. Inter-node communication is reduced by placing highly communicating tasks together. After that, T3-Scheduler places frequently communicating tasks in the same slot. As a result, the communication between the slots is reduced. T3-Scheduler is compared with Online Scheduler and R-Storm and results show that T3-Scheduler increases throughput by 32% for the two real-world applications on a 10-nodes cluster.

A basic problem in a stream processing system is the scheduling problem to minimize tuple processing time. To achieve this goal, Li et al. (2018) developed a model-free approach that learns from its experience. Deep Reinforcement Learning is introduced for model-free control which minimizes tuple processing time by learning the system environment using runtime statistics and making decisions under Deep Neural Networks. Extensive experiments show that the proposed framework reduces tuple processing by 33.5%. The proposed system is built on historical data; therefore, it suffers from a cold start problem. This work was compared with Actor-critic-based method, default scheduler, model-based method, and Deep Q Network-based method using three topologies on a cluster of 11 computing nodes.

Selecting suitable executors followed by an appropriate mapping between executors to nodes affects overall throughput and resource utilization. To overcome this effect, a heterogeneity-aware scheduler is proposed (Nasiri et al., 2020) that finds the proper number of executors and maps them to the most suitable node. The proposed algorithm scales up the topology’s DAG over a given cluster by increasing the topology input rate and generating new tasks from bottlenecked executors until an optimal solution is reached. When compared to the default scheduler of Storm (version 0.9.5), the proposed scheduler provides up to 44% throughput enhancement. A brute-force algorithm is used for scheduling which takes 18 hours for producing results which is a major drawback of this work. Some production applications like PageLoad topology, processing topology, and network monitoring topology are used to evaluate this work on a small network of four computing nodes.

The execution performance in Storm is strongly affected by the strategy of scheduling the topology’s components. Gulzar Ahmad et al. (2020) also proposed a two-phase strategy for scheduling Storm topologies. First phase partitions the topology’s DAG to minimize communication between partitions. The second phase allocates each partition on a single node which reduces the computational cost of inter-task communication of a partition to zero. Moreover, partial task duplication is also implemented to further reduce the execution time. Results have shown 20% improvement in average execution time as compared to the default scheduler. The major limitation of this work is that each partition is mapped to a single node. If available nodes are less than the required number then this scheduler will not work. Moreover, partial task duplication is overprovisioning which is also an overhead. Performance comparison of this work was made with default scheduler, R-Storm, and GA using EURExpressII workflow on a five nodes cluster.

Recently, Al-Sinayyid & Zhu (2020) proposed MT-Scheduler for stream processing which aims to maximize throughput on a heterogeneous cluster. It minimizes data communication and processing time to achieve the fastest frame rate for streaming applications. Dynamic programming is used to map the topology based on computing and communication requirements in a resource-aware fashion. First, the topology’s DAG is linearized using topological sort. Next, the critical path is identified using the polynomial longest path algorithm. Then, the mapping schema is determined for the topological tasks in the critical path. The non-CP tasks are mapped using a simple layer-oriented (greedy) method. The MT-Scheduler decreases latencies by 46% and improves throughput by 54% as compared to the storm scheduler in polynomial-time. The MT-Scheduler is implemented in Apache Storm (version 0.9.7) with a cluster of eight heterogeneous nodes and a comparison was made with the default storm and adaptive scheduler (Aniello, Baldoni & Querzoni, 2013).

Table 1 describes a summary of the literature review. The default storm scheduler makes executor’s assignment without considering communication traffic, which makes a significant impact on the performance (Xu et al., 2014). It also engages maximum nodes, regardless of workload (van der Veen et al., 2015). This over-provisioning may cause low resource utilization and higher communication cost. In addition, the application’s computational requirement is overlooked which reduces throughput (Madsen & Zhou, 2015; Peng et al., 2015; Eskandari, Huang & Eyers, 2016; Weng et al., 2017). The default Storm is designed for the homogeneous cluster which may introduce performance bottleneck problem (Liu, 2017; Smirnov, Melnik & Nasonov, 2017; Zhang et al., 2017). According to Table 2, nine times better throughput and 350% better CPU utilization have been achieved so far.

**Table 1:** Summary of literature review algorithms.

Reference	Scheduling aspects						
	Dynamic	Traffic-aware	Topology-aware	Heterogeneous	Self-adaptive	Resource-aware	Network-aware
(Gulzar Ahmad et al., 2020)	✗	✗	✓	✗	✗	✗	✗
(Al-Sinayyid & Zhu, 2020)				✓	✗	✓	✓
(Weng et al., 2017)		✓	✗	✗	✓	✓	✗
(Liu, 2017)			✓	✓	✗	✗	✗
(Xue et al., 2015)					✓	✓	✗
(Peng et al., 2015)	✓	✗	✗	✓	✗	✗	✓
(Qian et al., 2017)			✓	✗	✗	✗	✗
(Nasiri et al., 2020)				✓	✓	✗	✗
(Muhammad, Aleem & Islam, 2021)						✓	✗
(Fan, Chen & Hu, 2016)		✓	✗	✗	✓	✗	✗
(Li et al., 2018)							
(Aniello, Baldoni & Querzoni, 2013)				✓	✓	✗	✗
(Xu et al., 2014)							
(Liu & Buyya, 2018)						✓	✗
(Fischer & Bernstein, 2015)			✓	✗	✗	✗	✗
(Liu et al., 2017)							
(Eskandari, Huang & Eyers, 2016)					✓	✗	✗
(Eskandari et al., 2018b)				✓	✗	✓	✗
(Li & Zhang, 2017)					✓	✗	✗
(Muhammad & Aleem, 2021)						✓	✗

**Table 2:** Performance metric used in literature.

Reference	Performance metric		
	Resource utilization	Throughput	Latency
(Liu & Buyya, 2018)	-	+16.25%	-
(Gulzar Ahmad et al., 2020)		+21%	-
(Liu, 2017)		+24%	−20%
(Aniello, Baldoni & Querzoni, 2013)		+30%	-
(Eskandari et al., 2018b)		+32%	-
(Li et al., 2018)		+33.50%	-
(Smirnov, Melnik & Nasonov, 2017)		+40%	-
(Nasiri et al., 2020)		+44%	-
(Al-Sinayyid & Zhu, 2020)		+54%	−46%
(Fischer & Bernstein, 2015)		+56%	-
(Xu et al., 2014)		+84%	-
(Li & Zhang, 2017)		+91%	−50%
(Qian et al., 2017)		+1.25 times	−18.70%
(Liu et al., 2017)		+9 times	−9.80%
(Madsen & Zhou, 2015)		-	−20%
(Eskandari, Huang & Eyers, 2016)			−50%
(Xue et al., 2015)			−55.90%
(Weng et al., 2017)	+15% CPU +60% Memory	-	-
(Fan, Chen & Hu, 2016)	−3% extra resources used	+67%	
(Peng et al., 2015)	+350% CPU utilization	+50%	

After a detailed critical analysis of the existing research work done in this domain, it is desirable to have a dynamic resource-aware scheduler that can distribute jobs among heterogeneous cluster to improve resource utilization. Moreover, it can assign workload based on data communication to achieve maximum throughput and reduce data communication between components in a network-aware fashion.

## Mf-storm scheduler

The MF-Storm scheduler maps Storm topology (weighted acyclic graph of computation) on a heterogeneous cluster with improved resource utilization and increased throughput. MF-Storm maps computational requirements (processing and communication requirements) of topology on the available network of workers (with a given processing and communication power) in an optimal way to maximize the average throughput per node of the cluster against the given topology.

MF-Storm schedule the jobs in two phases. The first phase, termed Graph Partitioning, formulates a weighted directed acyclic graph (W-DAG) from the topology with intra-executor (spout and bolt) computation requirements and inter-executor communication requirements. The graph is then processed by the max-flow min-cut algorithm which gives us limiting communication links in the network and maximum flow which can be achieved with the limiting links. The limiting links can then be used to distribute the group of executors between different workers of the cluster. Groups of frequently communicating executors are assigned to the nearby workers to decrease inter-worker communication.

The second phase, Physical Mapping, intelligently maps to a pool of workers by assigning all the group of executors to a worker based on the worker’s computation power and reduced communication need as per the modeled W-DAG. A group with maximum processing requirement can be assigned to a worker with higher computational power.

The Algorithm 1 is a driver program for the proposed MF-Storm scheduler. The algorithm gets unassigned jobs as input and produces the physical map for scheduling. Initially, with the help of the topology’s code, the topology’s DAG is retrieved which contains the connectivity between the executors. The MF-Storm needs inter-executor connectivity as well as inter-executor traffic to generate an execution plan. Therefore, Inter-executor traffic is retrieved from the traffic log to generate modeled DAG (line 3) as W-DAG. Once we have the executor’s connectivity as well as traffic the next step is to partition the graph into sub-graphs. These sub-graphs are created to have maximum intra-graph communication and minimum inter-graph communication to enhance throughput. To achieve this goal, a Graph partitioning algorithm is used known as Max-flow_Min-cut (line 4). Finally, for physical mapping of partitions to workers, Map_Partitions_To_Workers is invoked (line 5).

**Algorithm 1:** MF-Storm Scheduler

Input: Unassigned Topologies (T[ ])
Output: Topologies to Cluster Nodes Physical Mapping
1 while all t in T[ ] are not assigned do
2 DAG ← get_Topology_DAG(t)
3 W_DAG ← get_Modeled_DAG(DAG)
4 Graph_Partitions ← max-Flow_Min-Cut(W_DAG)
5 Physical_Mapping ← map_Partitions_To_Workers(Graph_Partitions)
6 return Physical_Mapping

Once executors are partitioned into groups, the next step is to map partitions to workers in a resource-aware manner. First, Map_Partitions_To_Workers (as shown in Algorithm 2) calculates the computation power in Floating Point Operations Per Second (FLOPs) for each node applying Eq. (1) (lines 2–6). All machines are positioned in terms of FLOPs to handle heterogeneity (Khalid et al., 2018). FLOPs represent the number of calculations a processor can perform in a second (“FLOPS (Floating Point Operations Per Second) Definition”, https://techterms.com/definition/flops).

**Algorithm 2:** Map_Partitions_To_Workers

Input: Graph_Partitions
Output: Physical_Mapping
1 ClusterNode Nodes ← ClusterInfo.getNodes()
2 foreach n ∈ Nodes do
3 cores ← n.getCores()
4 frequency ← n.getFrequency()
5 flops ← n.getFLOPs()
6 n.GFLOPS ← cores x frequency x flops
7 Nodes ← Nodes.Sort(‘Desc’)
8 Graph_Partitions ← Graph_Partitions.Sort(‘Desc’)
9 foreach p ∈ Graph_Partitions do
10 if n.FreeSlots equals to 0 then
11 n = Nodes.getNextNode()
12 Physical_Mapping.add(p, n)
13 decrement n.FreeSlots to 1
14 return Physical_Mapping

(1) $$ProcessingSpeed = \; \; NumberOfCores\; \times \; \displaystyle{{cycle} \over {second}}\; \times \; \displaystyle{{flops} \over {cycle}}$$

After FLOPs calculation, the nodes are sorted in descending order with respect to their computing capacity (line 7). Also, graph partitions are sorted in descending order with respect to their inter-executor communication (line 8). Lastly, the partition with the highest communication traffic is allocated to the most powerful worker. Partitions are distributed to workers once all workers are used then the next machine is occupied (lines 9–13). With this approach, minimum machines are used, and inter-machine communication is also decreased.

## Experimental evaluation

A heterogeneous cluster is designed with one nimbus node along with ZooKeeper, and four supervisors to assess the proposed MF-Storm scheduling scheme. Ubuntu version 19.04 is installed on each node along Storm 2.0.1 (https://github.com/apache/storm) coordinated by Zookeeper 3.4.13, with zeromq, JZMQ, and Python. To achieve heterogeneity, the system configuration has high-computational nodes equipped with eight cores whereas the low-capacity nodes have four and two cores. Table 3 contains configurations of the cluster used for experiments.

**Table 3:** Hardware configurations of the cluster used for experiments.

Machine name	Processor configuration	FLOPs/cycle	GFLOPs	GFLOPs index
Nimbus-Server Zookeeper-Server	Intel Core i7 3.40 GHz × 8	6816	185395	1
Node-D	Intel Core i7 3.40 GHz × 8	6816	185395	1
Node-C	Intel Core i7 3.40 GHz × 6 (2 Cores are disabled)	6816	139046	2
Node-A	Intel Core i7 3.40 GHz × 4 (4 Cores are disabled)	6816	92698	3
Node-B	Intel Core i5 3.20 GHz × 4	6385	81728	4

### Performance evaluation

The Apache storm ships with multiple schedulers (“Apache Storm Scheduler”, http://storm.apache.org/releases/2.0.0/Storm-Scheduler.html) *e.g*. Default Scheduler, Isolation Scheduler, Resource-aware Scheduler, and Multitenant Scheduler. The first three follow single-tenant architecture while the last one uses multitenant architecture. MF-Storm is derived from Default Scheduler which is based upon single-tenant architecture. Therefore, MF-Storm is compared with the first three single-tenancy schedulers.

- **The default scheduler** (Liu, 2017)
- **R-Storm** (Peng et al., 2015)
- **Isolation scheduler** (GitHub, 2014)

The Storm has various types of topology structures *e.g*., linear, diamond, star *etc.* The performance of the MF-Storm is assessed with the help of four linear topologies. We have selected topologies that can process data and make a decision. In this context, the topologies which are shipped with Apache Storm suit (“Example Storm Topologies”, https://github.com/apache/storm/tree/master/examples/storm-starter) and used by researchers as a benchmark are the following:

- **Word Count Topology** (“Storm Topology Explained using Word Count Topology Example | CoreJavaGuru”, http://www.corejavaguru.com/bigdata/storm/word-count-topology)
- **Exclamation Topology** (Karanth, 2014; GitHub, 2019)

Two more topologies are developed by modifying the above topologies which are as follows:

- **Tweet Word Count Topology** that breaks 5,000 tweets into words and then counts the number of occurrences of each word. The actual Word Count Topology is generating words with a 100-millisecond delay which is reduced to 50 to see the impact;
- **Tweet Exclamation Topology** that emits a random word from the collection of 2,500 words and then appends three exclamation marks (!!!) to the words. The actual Exclamation Topology is generating words with a 100-millisecond delay which is reduced to 50 to see the impact.

### Evaluation metrics

In the literature, similar nature of work is being evaluated using throughput, latency, and/or resource utilization. In this article, throughput and resource utilization are used for evaluation.

- **Throughput:** represents the number of tuples processed per unit time

- **Resource utilization** represents the number of supervisor nodes used for job execution. For example, a supervisor node has five workers, and even if a single worker is being used in topology execution then in this work, it will be considered a used resource.

We think that only throughput does not truly represents the efficiency of a scheduler. To depict the impact of both measures, an evaluation matrix named as average throughput per node (Eq. (3)) is used in this work.

(2) $$AverageThroughput = TotalTuplesProcessed \div TotalTimeTaken$$

(3) $$AverageThroughputPerNode = AverageThroughput \div TotalNodesUsed$$

### Result assessment

The assessment of the MF-Storm using four topologies with three state-of-the-art schedulers is presented in this section. All four topologies were executed with the three selected schedulers under different scenarios with a varying number of required worker processes (slots). Likewise, the available worker process for each machine varied, and measurements were taken to analyze the effect on throughput in all the scenarios as shown in the following Table 4.

**Table 4:** Different scenarios for the experiments.

	Scenario 1	Scenario 2	Scenario 3	Scenario 4	Scenario 5	Scenario 6
Supervisor nodes	4					
Available slots per node	1			2		3
Total available slots for topology execution	4 (4 nodes × 1 slot)			8 (4 nodes × 2 slots)		12 (4 nodes × 3 slots)
Slots required for topology execution	1	2	3	2	3	3

We have used different scenarios for experiments. Scenario 1 says that we have one slot per node and we have four nodes in the cluster. So, we have four (1 slot × 4 nodes) slots available for topology execution. If one slot is required then one slot from these four slots will be selected by the scheduler. This slot’s choice will vary from the scheduler to the scheduler based on their algorithm. For a reasonable comparison, each experiment is run for 20–25 min while reading is recorded at a 60-s interval.

### Comparison with other schedulers

#### Word count topology

**Figure 1:**
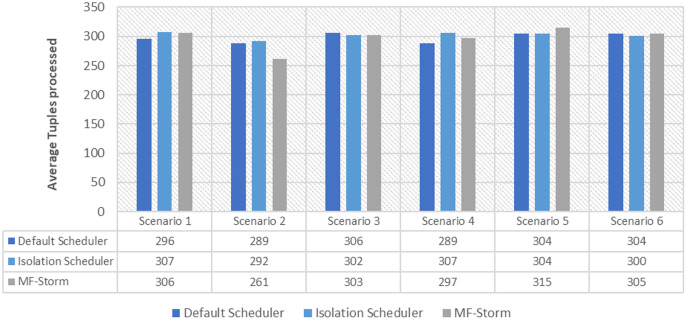
Average throughput calculated using Eq. (2) for word count topology.

Figure 1 shows the average throughput produced using scenarios 1 to 6 with the y-axis standing for average tuples processed in 20–25 min (throughput). Experiments run for hours, however, the performance after around 5 min stays the same.

**Figure 2:**
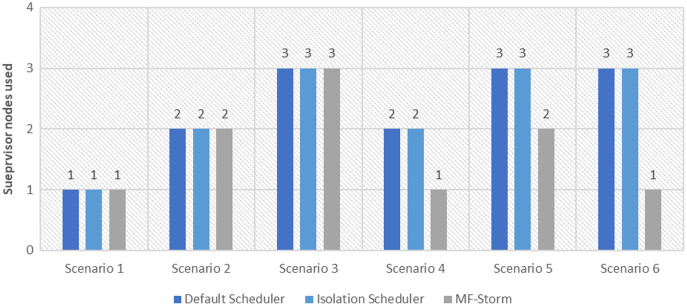
The number of nodes used for word count and tweet word count topology by scheduling algorithms.

**Figure 3:**
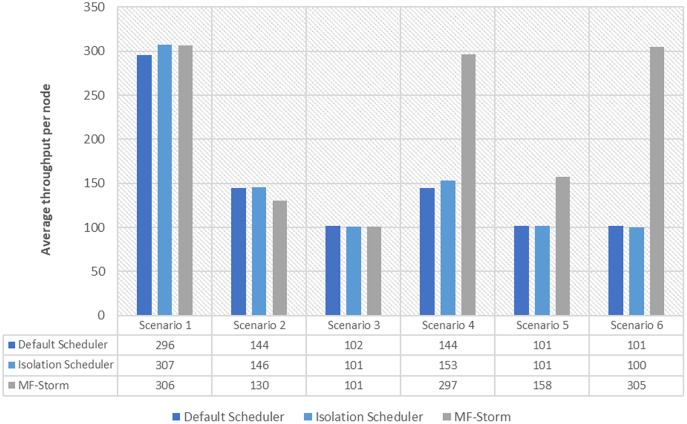
Average throughput per node for word count topology calculated using Eq. (3).

In Fig. 2, the total number of nodes used by each scheduling algorithm is presented. The average throughput per node for word count topology under scenarios 1 to 6 is calculated using Eq. (3) (see Fig. 3). Here, MF-Storm gains a maximum of 201% on average throughput per node with respect to the default scheduler and 205% as compared to the Isolation scheduler for scenario 6.

#### Exclamation topology

**Figure 4:**
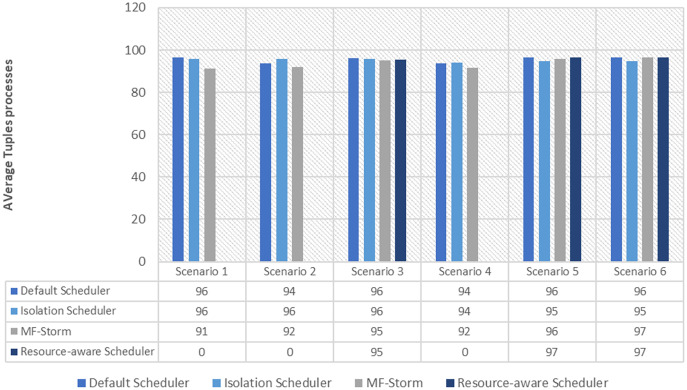
Average throughput calculated using Eq. (2) of the scheduling algorithms for exclamation topology.

**Figure 5:**
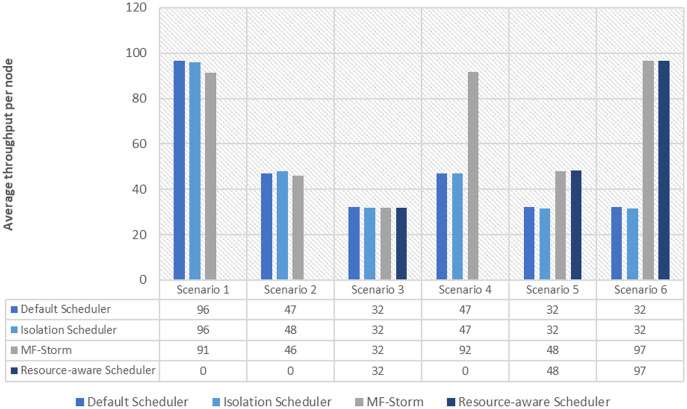
Average throughput per node for exclamation topology calculated using Eq. (3).

**Figure 6:**
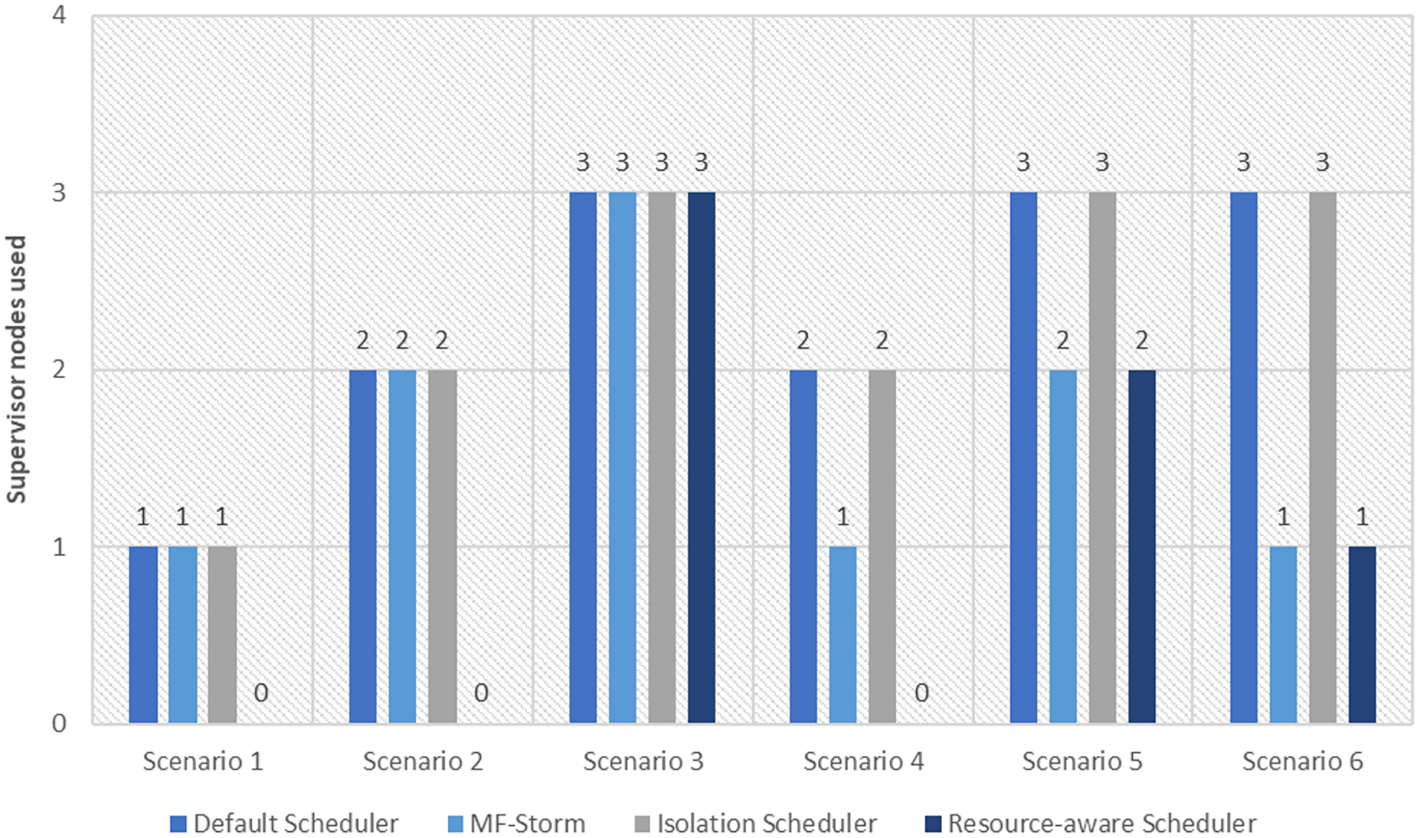
The number of nodes used for exclamation and tweet exclamation topology by scheduling algorithms.

Similar experiments have been performed for exclamation topology as well. Figure 4 shows the average throughput calculated using Eq. (2) of the state-of-the-art algorithms for exclamation topology for different scenarios. With the help of Eq. (3), the average throughput per node for MF-Storm and baseline scheduling algorithms are calculated as shown in Fig. 5. Improvement in average throughput per node is obtained 200% and 205% for MF-Storm to the default scheduler and isolation scheduler. MF-Storm achieves these results with 33% of resources concerning the other schedulers which acquire three nodes for execution (see Fig. 6). The reason is that MF-Storm consumes all available slots of a machine and then uses the next machine. Conversely, the default Storm uses a round-robin approach.

#### Tweet word count topology

**Figure 7:**
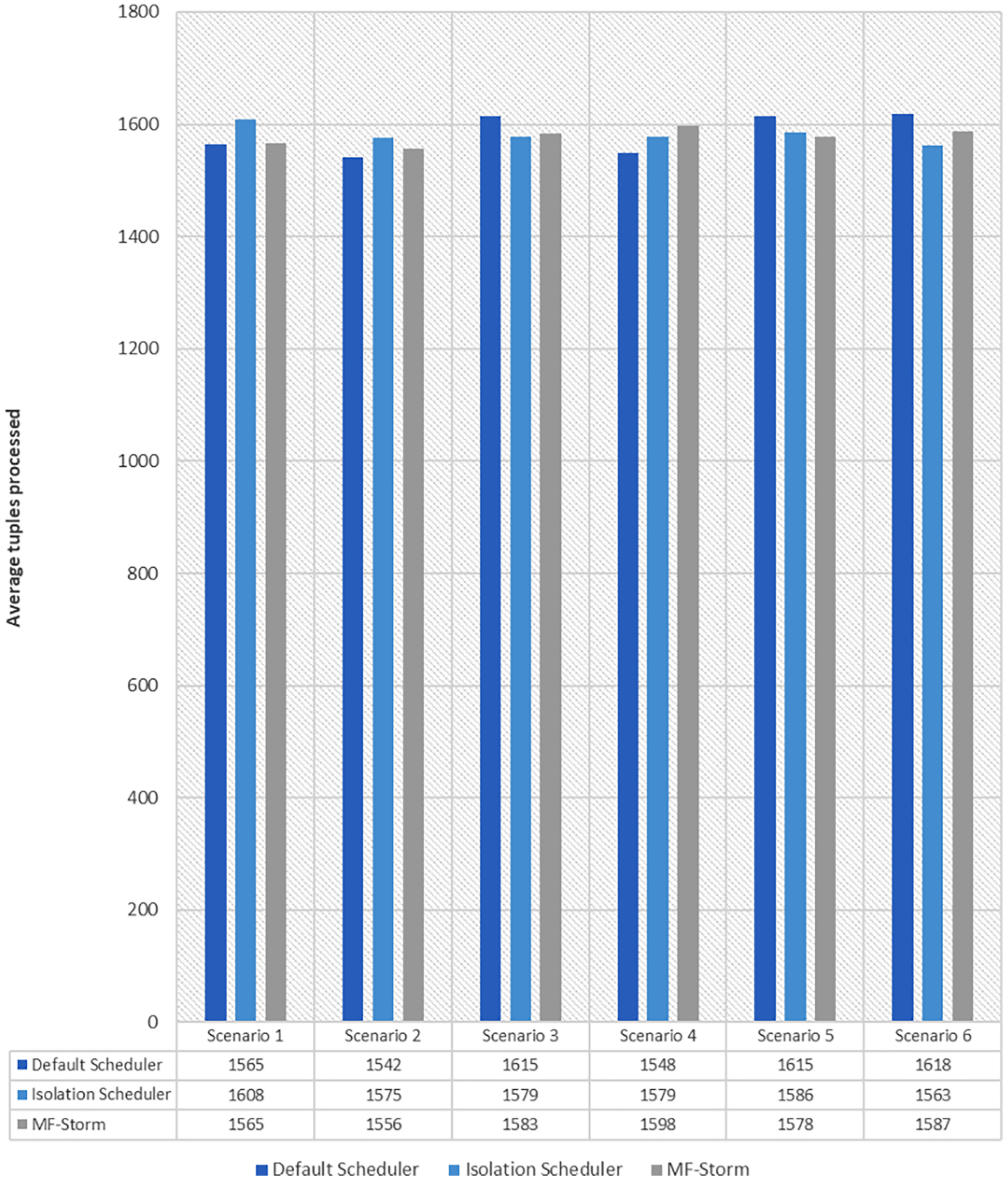
Average throughput calculated using Eq. (2) for tweet word count topology.

**Figure 8:**
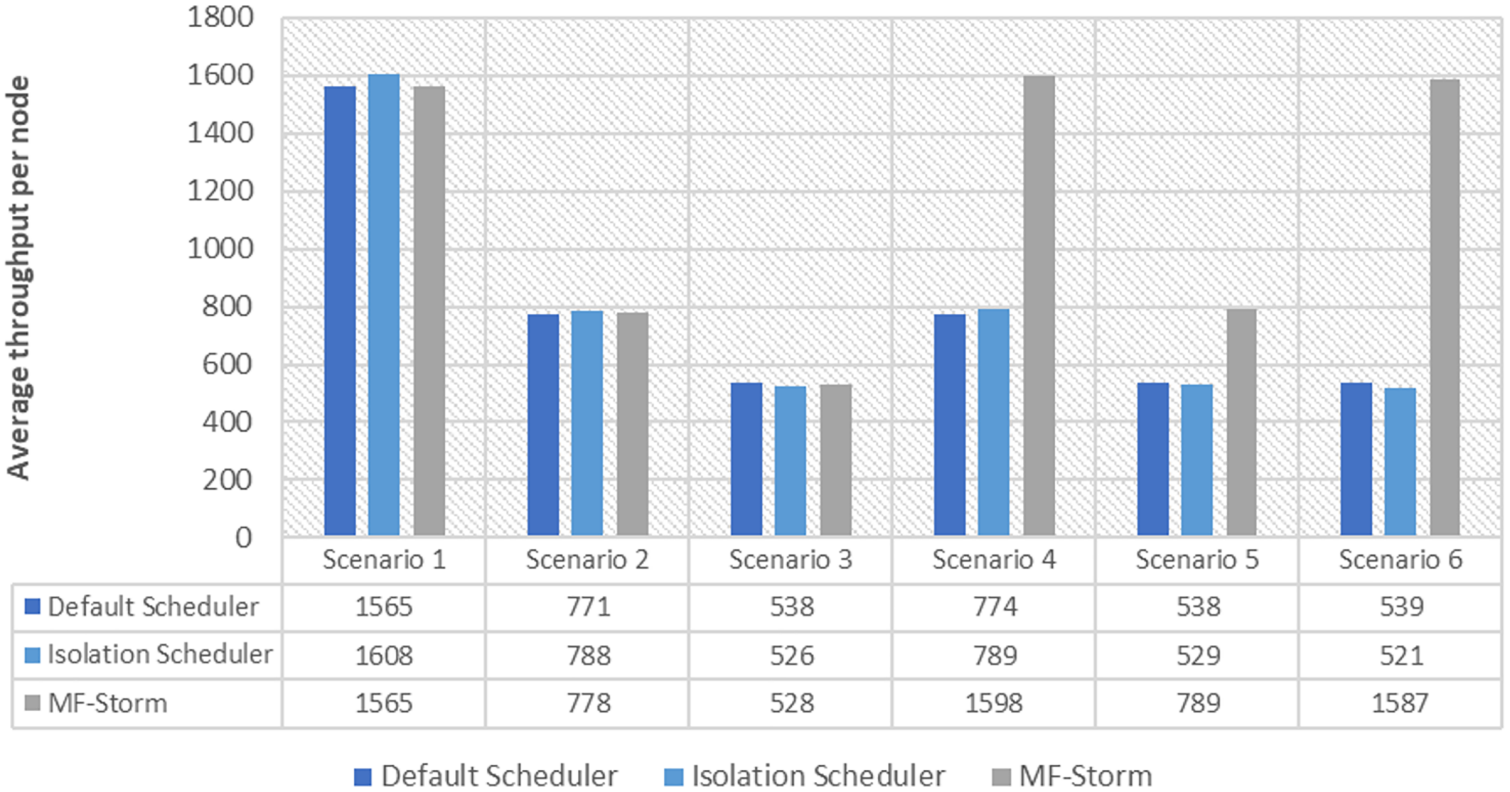
Average throughput per node for tweet word count topology calculated using Eq. (3).

The same procedure is repeated for tweet word count topology. Figure 7 shows communication traffic produced using scenarios 1 to 6. MF-Storm attained a maximum of 194% and 205% improvement in average throughput per node (Fig. 8) for scenario 6 as compared to the default Storm and isolation scheduler with 66% fewer resources (see Fig. 2).

#### Tweet exclamation topology

**Figure 9:**
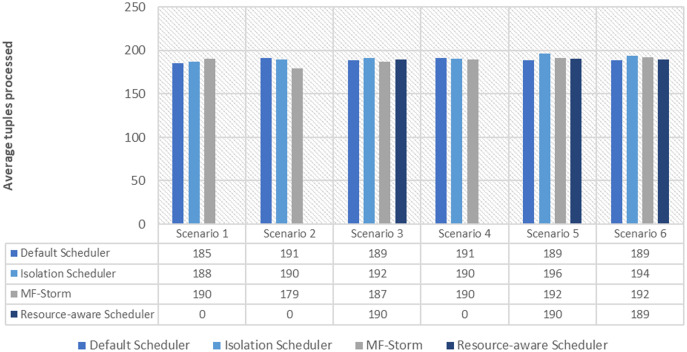
Average throughput calculated using Eq. (2) of the scheduling algorithms for tweet exclamation topology.

The comparison of MF-Storm with other schedulers for tweet exclamation topology under scenarios 1 to 6 is shown in Fig. 9. MF-Storm achieved 206%, 198%, and 2% improvement when compared to the default, isolation, and resource-aware schedulers. MF-Storm achieves this improved throughput with 66% less computational resources as compared to the others that employ 100% available resources (Fig. 6).

#### Result discussion

From the results, it is clear that MF-Storm performs well in average throughput per node and resource usage when compared to the other scheduling algorithms. It will be difficult to achieve these results if either communication between executors or available resources are ignored. The default Apache Storm scheduler uses a round-robin approach which may increase inter-node communication; hence resulting in decreased throughput. Similarly, it occupies maximum computing nodes in the cluster. Conversely, MF-Storm consumes all available slots of a node then it moves to the next node. In this way, a compact slots assignment is achieved which is directly proportional to higher throughput. For configurations where fewer resources are employed, the difference in average throughput is also minimal and *vice versa*.

**Figure 10:**
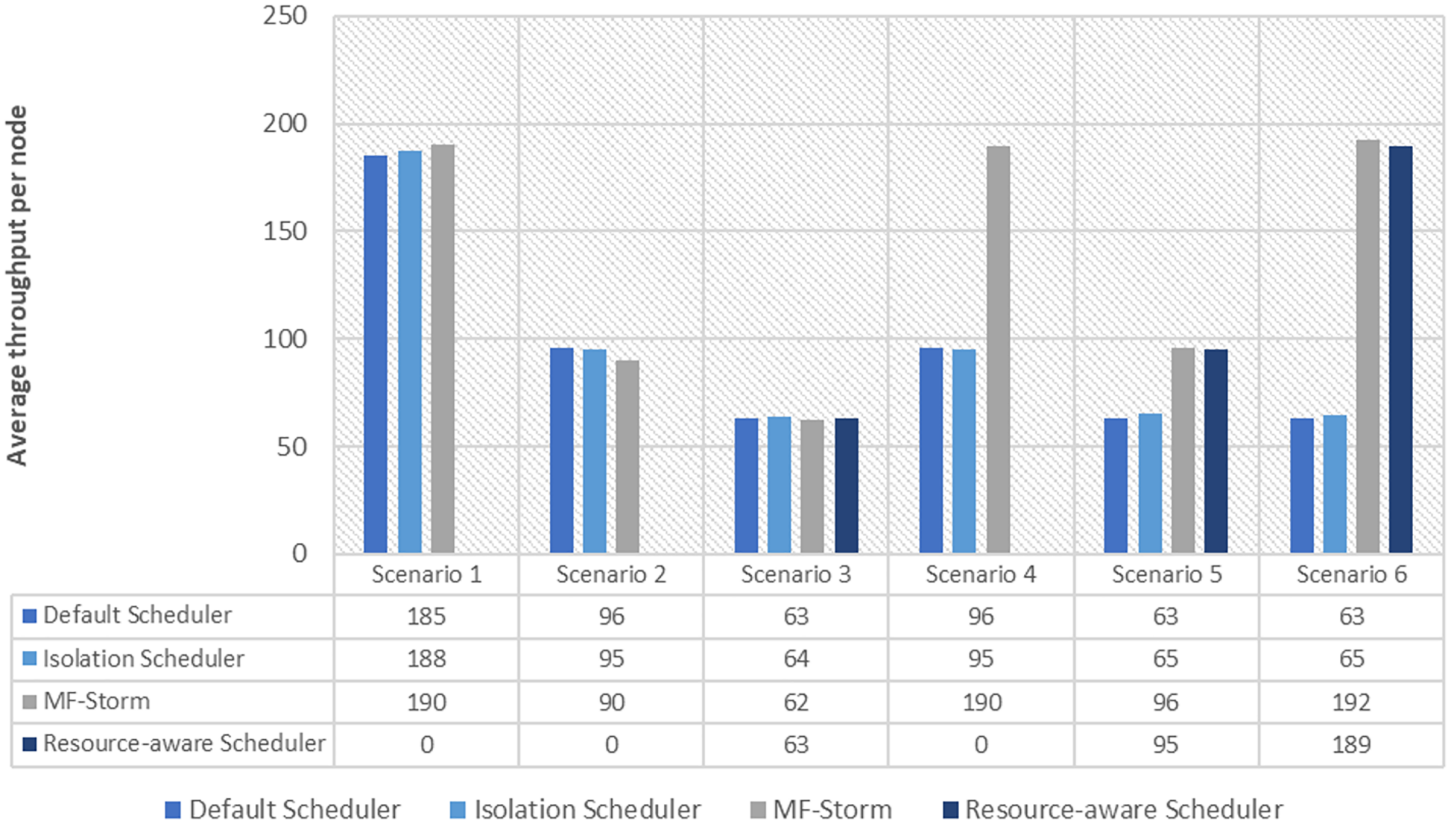
Average throughput per node for tweet exclamation topology calculated using Eq. (3).

The Isolation scheduler executes topologies in isolation on a dedicated cluster. The isolation scheduler generates a different execution plan as compared to the default scheduler. The focus of this scheduler is to maximize cluster usage which increases inter-node communication and adversely affects throughput. It has been shown (in Figs. 1–10) that the isolation scheduler performed worst for cluster configurations (see Table 3).

R-Storm (Peng et al., 2015) increases throughput by maximizing resource utilization but the major weakness of R-Storm is that it overlooks the required number of slots given by the user at the time of topology submission. Secondly, If the required number of slots is not available, then R-Storm does not run the job. Similarly, the user cannot direct R-Storm to use slots other than calculated by R-Storm. That is why we do not have results for word count and tweet word count topologies for R-Storm because three slots are needed for topology execution and R-Storm executes these topologies in five slots.

Table 2 shows the results generated by different schedulers proposed in this domain. Weng et al. (2017) considered CPU and Memory while scheduling. Only 15% improvement in CPU use is achieved; however, MF-Storm saved up to 66% computational resources of the cluster. Similarly, with the help of over-provisioning (Fan, Chen & Hu, 2016); reached 67% better throughput. Comparatively, MF-Storm with one-third of computational resources made up to 206% enhancement in average throughput per node with respect to the default scheduler.

As mentioned in the “Experimental evaluation” section, we have performed experiments using three state-of-the-art schedulers under six different scenarios. If we calculate the average percentage improvement obtained by word count topology for the default scheduler, then it will be 159%. Similarly, 157% average percentage improvement is recorded for the isolation scheduler. On average, 158% (Fig. 3) improvement factor is achieved by MF-Storm for word count topology as compared to both schedulers. Moreover, 138% (Fig. 5), 158% (Fig. 8), and 138% (Fig. 10) are scored for exclamation, tweet word count, and tweet exclamation topology, respectively. Therefore, the average improvement factor for all topologies for MF-Storm is 148% using 30% fewer computational resources.

## Conclusions and future work

In this article, we presented a Storm scheduler using topology’s traffic and cluster computation power. First, with the help of a graph algorithm, the topology’s DAG was partitioned. After that, these partitions were sorted in descending order according to their communication pattern. Then partitions were assigned to the most powerful machine with respect to the machine’s FLOPs in the heterogeneous cluster and so on. In this way, powerful machines were utilized first resulting in improved throughput. The compact scheduling strategy adopted by MF-Storm enhanced resource utilization with reduced inter-task communication. As a result, the average improvement factor for all topologies for MF-Storm was 148% using 30% fewer computational resources. In this article, we considered four different jobs for scheduling. For evaluation, we used linear topologies and in future work, we intend to experiment using other extensive topology types to gauge the scheduling capabilities of the MF-Storm.

## Supplemental Information

10.7717/peerj-cs.1077/supp-1 Supplemental Information 1 Random Sentence Spout. Word count topology uses this file to generate sentences.

10.7717/peerj-cs.1077/supp-2 Supplemental Information 2 Test word spout. Exclamation topology uses this file to generate words.

10.7717/peerj-cs.1077/supp-3 Supplemental Information 3 Proposed scheduler source code in java.

10.7717/peerj-cs.1077/supp-4 Supplemental Information 4 Word Count Topology. Two different jobs are used in this work for the sake of testing the proposed work. *E.g*., Word Count Topology and Exclamation Topology. These jobs generated random sentences as raw data with the help of java code.

10.7717/peerj-cs.1077/supp-5 Supplemental Information 5 Exclamation Topology. Two different jobs are used in this work for the sake of testing the proposed work. *E.g*., Word Count Topology and Exclamation Topology. These jobs generated random sentences as raw data with the help of java code.

## Additional Information and Declarations

### Data Availability

The following information was supplied regarding data availability: The code is available in the Supplemental Files.
